# Non-Invasive Optical Imaging of Eosinophilia during the Course of an Experimental Allergic Airways Disease Model and in Response to Therapy

**DOI:** 10.1371/journal.pone.0090017

**Published:** 2014-02-25

**Authors:** M. Andrea Markus, Christian Dullin, Miso Mitkovski, Eva Prieschl-Grassauer, Michelle M. Epstein, Frauke Alves

**Affiliations:** 1 Department of Haematology and Oncology, University Medical Center Göttingen, Göttingen, Germany; 2 Department of Diagnostic and Interventional Radiology, University Medical Center Göttingen, Göttingen, Germany; 3 Light Microscopy Facility, Max-Planck-Institute of Experimental Medicine, Göttingen, Germany; 4 Marinomed Biotechnologie GmbH, Vienna, Austria; 5 Department of Dermatology, Division of Immunology, Allergy and Infectious Diseases, Experimental Allergy, Medical University of Vienna, Vienna, Austria; 6 Department of Molecular Biology of Neuronal Signals, Max-Planck-Institute of Experimental Medicine, Göttingen, Germany; Murdoch University, Australia

## Abstract

**Background:**

Molecular imaging of lung diseases, including asthma, is limited and either invasive or non-specific. Central to the inflammatory process in asthma is the recruitment of eosinophils to the airways, which release proteases and proinflammatory factors and contribute to airway remodeling. The aim of this study was to establish a new approach to non-invasively assess lung eosinophilia during the course of experimental asthma by combining non-invasive near-infrared fluorescence (NIRF) imaging with the specific detection of Siglec-F, a lectin found predominantly on eosinophils.

**Methodology/Principal Findings:**

An ovalbumin (OVA)-based model was used to induce asthma-like experimental allergic airway disease (EAAD) in BALB/c mice. By means of a NIRF imager, we demonstrate that 48 h–72 h after intravenous (i.v.) application of a NIRF-labeled anti-Siglec-F antibody, mice with EAAD exhibited up to 2 times higher fluorescence intensities compared to lungs of control mice. Furthermore, average lung intensities of dexamethasone-treated as well as beta-escin-treated mice were 1.8 and 2 times lower than those of untreated, EAAD mice, respectively and correlated with the reduction of cell infiltration in the lung. Average fluorescence intensities measured in explanted lungs confirmed the *in vivo* findings of significantly higher values in inflamed lungs as compared to controls. Fluorescence microscopy of lung cryosections localized the i.v. applied NIRF-labeled anti-Siglec-F antibody predominantly to eosinophils in the peribronchial areas of EAAD lungs as opposed to control lungs.

**Conclusion/Significance:**

We show that monitoring the occurrence of eosinophils, a prominent feature of allergic asthma, by means of a NIRF-labeled antibody directed against Siglec-F is a novel and powerful non-invasive optical imaging approach to assess EAAD and therapeutic response in mice over time.

## Introduction

Allergic asthma is a chronic inflammatory disease of the lungs, which is characterized by a variable degree of bronchial obstruction, airway hyperresponsiveness (AHR) and increased mucus production. With over 300 million people affected and this number growing steadily, asthma is still a major health issue. While mild to moderate asthma is relatively well controlled by glucocorticoid therapy [1], 5–10% of asthmatics are difficult to treat with current therapies and warrant a continuing search for new drugs [2]. Similar to other complex and heterogeneous diseases, our understanding of asthma is slowed by the fact that both genetic as well as environmental factors contribute to its origin and progression, and by the variety of cellular and molecular pathways involved [3]. As a result, animal models, especially in mice, have been vital in improving our knowledge of asthma and the development and validation of novel treatments [4]. Many of the characteristic features of human atopic asthma can be seen in mouse models. For example, following allergen challenge, profound eosinophilic infiltration of lung tissue and airways, an increase of lymphocytes, neutrophils, and monocytes in the lungs, activation of alveolar macrophages and thickening of the airway epithelium with a marked goblet cell hyperplasia are all characteristics found in both humans and mice [5].

Until recently, preclinical animal studies, including the assessment of mouse EAAD, relied heavily on invasive or terminal procedures such as bronchoalveolar lavage (BAL) and histology of excised tissue. Latest improvements of imaging techniques such as PET, SPECT, MRI, CT and OCT have advanced non-invasive research on pulmonary diseases [6]. However, these techniques mainly facilitate the anatomical or structural assessment of the diseased lung and/or make use of radioactive agents. Optical imaging poses a great advantage, offering a rapid, cheap and easy methodology, which enables the detection of specific targets in a live animal over time [7]. Presently, near infrared fluorescent (NIRF) probes revealed several benefits over other fluorescent dyes because they minimize autofluorescence and penetrate deeper into the tissue [8]. Importantly, NIRF imaging lacks radioactivity and is therefore considered an alternative to nuclear imaging, the current gold standard for clinical functional imaging.

However, molecular imaging of lung diseases and in particular allergic asthma using fluorescence imaging (FI) is limited [6] and unspecific [9], [10]. Only proteinases such as matrix metalloproteinases (MMPs) and cathepsins [9], [10] as well as selectins [11] have so far been targeted with smart probes. However, such optical sensors may detect inflammation unrelated to eosinophilia. We took a new, more specific, approach to detect the allergic inflammatory process underlying asthma by targeting Siglec-F, a member of the family of Siglecs (sialic acid-binding, Ig-like lectins), which are single-pass transmembrane cell surface proteins found predominantly on leucocytes [12]. Siglec-F is a functional paralog of the human Siglec-8, both proteins preferentially recognising a sulphated glycan ligand closely related to sialyl Lewis X, a common ligand for the selectin family of adhesion molecules [12]. Most siglec proteins undergo endocytosis, an activity tied to their roles in cell signaling and innate immunity. Both, the human as well as the mouse protein, are specifically upregulated on eosinophils during allergic inflammation, and therefore, represent specific markers for detection of allergic reactions, involving eosinophils. Induction of allergic lung inflammation in mice causes up-regulation of Siglec-F on blood and bone marrow eosinophils as well as quantitative up-regulation of endogenous Siglec-F ligands in the lung tissue and airways [13]. A weaker expression was also reported on macrophages [13], [14]. The recruitment of eosinophils to the airways occurs at the late-phase of allergic inflammation and their release of proteases and proinflammatory factors is thought to eventually lead to airway remodeling [15]. Eosinophilia is, therefore, an excellent marker for monitoring allergic inflammation. It was recently shown that anti-Siglec-F alone or in combination with anti-CD45 can be used for the quantitative detection of eosinophils in mouse bone marrow and spleen and that the antigen profile CD45(+)SiglecF(+)CD11c(−) was the most effective at detecting eosinophils in the lung and correlated with direct morphometric counts under all conditions evaluated [16].

We show here, that 2D fluorescence reflectance imaging (FRI) in combination with a NIRF-labeled antibody to Siglec-F, is an ideal technique to specifically monitor allergic lung inflammation *in vivo* and to evaluate the effect of therapeutic drugs in preclinical studies. We observed significantly higher fluorescence signal intensities over the lungs in mice with EAAD than in controls. Moreover, we non-invasively demonstrate decreased Siglec-F fluorescence signals over the lung in response to two different asthma therapies, the commonly used glucocorticoid dexamethasone, as well as beta-escin, a new anti-inflammatory drug derived from Chinese horse chestnut seeds.

## Materials and Methods

### Materials

Monoclonal rat anti-mouse-Siglec-F antibody and a rat IgG2a isotype control were purchased from BD Biosciences (Heidelberg, Germany). Siglec-F antibody was custom-labeled by Squarix Biotechnology (Marl, Germany) with either Alexa Fluor 750 (dye to protein ratio 2.8) or Alexa Fluor 680 (dye to protein ratio 4.5) (Life Technologies GmbH, Darmstadt, Germany). These NIRF-labeled anti-Siglec-F antibodies are designated anti-SiglecF-750 and anti-SiglecF-680, respectively. IgG2a isotype control antibody was labeled with Alexa Fluor 750 (dye to protein ratio 3.1).

### Animals

Pathogen-free female BALB/c mice, 6–8 weeks of age were purchased from Charles River Laboratories Inc. (Wilmington, MA). All animals were housed in a controlled environment with a regular 12-hour dark:light cycle, at 22°C and were fed laboratory chow (SAFE, Augy, France) and tap water ad libitum. Seven days before the imaging experiments, the food was switched to chlorophyll-free chow (Scientific Animal Food & Engineering, Augy, France) to reduce autofluorescence from the stomach and gut of the animals.

### Induction of EAAD and Treatment Schedule

**Figure 1:**
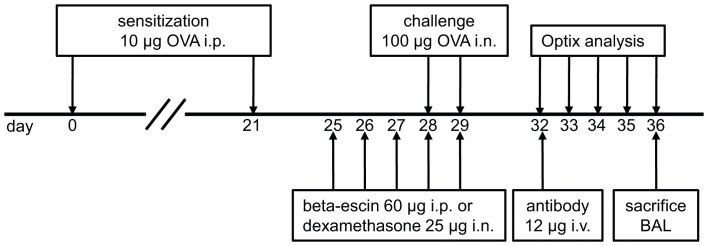
Induction of EAAD and treatment schedule. Schematic depiction of the experimental protocol used for the induction of EAAD, the application of treatments and the optical imaging performed.

As shown in Figure 1, BALB/c mice were immunized via intraperitoneal (i.p.) injection on days 0 and 21 with 10 µg OVA (Sigma-Aldrich) in a volume of 0.2 ml phosphate-buffered saline (PBS) per mouse. On days 28 and 29 post-immunization, mice were challenged intranasally (i.n.) by pipetting 25 µl of 100 µg OVA in PBS, into each nostril. Control mice received PBS only. Anti-Siglec-F-NIRF-labeled antibodies (12 µg in 150 µl PBS) were given either 3 or 4 days post challenge by tail vein injection and mice were repeatedly scanned over a given period of time by optical imaging. All intravenous (i.v.) injections and scanning procedures were performed under 2% isoflurane, 2l/min oxygen anesthesia for a maximum time of 20 min. The mice were sacrificed with an overdose of isoflurane after the last scan.

For treatment response studies, mice received either 25 µg dexamethasone (Sigma-Aldrich, Hamburg, Germany) in 50 µl PBS i.n. or 60 µg beta-escin in 200 µl PBS (Marinomed, Vienna, Austria) i.p. once a day from day 25 to 29. All i.n. procedures were performed under mild 2% isoflurane, 2l/min oxygen anesthesia of the mice.

### In vivo Optical Imaging

To decrease autofluorescence, BALB/c mice were shaved and chemically depilated (Isana depilation crème, Rossmann) to remove the fur from thorax and abdomen. Optical imaging was performed by FRI using the Optix MX2 System (ART, Montreal, Canada), which comprises an interface for inhalation anesthetics and four pulsed lasers (635, 670, 730 and 785 nm). During *in vivo* scans, mice were anaesthetized by inhalation with 2% isoflurane, 2l/min oxygen for 15–20 min per scan. For detailed description of the working principle of the Optix MX2 please refer to Dullin et al. [17].

OVA-challenged and control mice were prescanned to measure the autofluorescence signals of the animals. Three to four days post challenge, the animals were injected intravenously (i.v.) with either 12 µg of anti-SiglecF-750 antibody (n = 8 for EAAD; n = 6 for controls), anti-SiglecF-680 antibody (n = 5 for EAAD; n = 4 for controls) or 750-labeled rat IgG2a isotype control (n = 5) in 150 µl PBS and scanned at given time points. Alexa Fluor 750 fluorescence was measured using an excitation of 730 nm in combination with a 770 nm long-pass emission filter. Alexa Fluor 680 fluorescence was measured using an excitation of 670 nm in combination with a 700 nm long-pass emission filter. Scans were performed with a 1.5 mm (whole body scans) or 1.0 mm (lung scans) raster, a photon collection time (integration time) of 0.3–1 s per scan point and varying laser power. Intensity data and lifetime were analyzed with the OptiView-2-02-00 software (ART).

Fluorescence intensity data are displayed in normalized counts (NC), where the measured fluorescence intensity (counts) was normalized for varying laser power and integration times, allowing comparison of measurements with different settings. Data were quantified as average fluorescence intensity over a certain area of interest and subsequently corrected for autofluorescence by subtracting the average fluorescence intensity from the same region of interest in the respective prescans, as well as corrected for the dye to protein ratio of the different conjugates.

### Bronchoalveolar Lavage (BAL)

Following imaging (72 h after antibody injection), mice were sacrificed with an overdose of isoflurane. BAL was performed by washing the airways gently three times with 500 µl of 2% FCS/PBS after exposing and cannulating the trachea. Volumes were pooled and then washed once in the same buffer. Recovered cells were counted in a haemocytometer and 3×10^4^ cells were used for cytospins followed by Giemsa staining (Sigma Aldrich, Munich, Germany) for differential cell counting. Where indicated, cytospins were immunostained and counterstained with DAPI (4 µg/ml) for visualization of nuclei.

### Immunohistochemistry and Immunofluorescence

Explanted lungs were cannulated and filled with 600 µl of Tissue Tek OCT compound (Sakura Finetek Germany GmbH, Staufen, Germany) and immediately frozen in liquid nitrogen at −80°C. Frozen lung sections of 5 µm from untreated EAAD and control mice were cut on a Jung Frigocut 2800E cryostat microtome (Leica Microsystems, Wetzlar, Germany) and stained with monoclonal rat-anti-mouse-Siglec-F (BD Biosciences, Heidelberg, Germany) at 10 µg/ml in antibody-diluent (DAKO) and 4°C, overnight (o.n.). Subsequently, sections were incubated with an anti-rat-biotinylated secondary antibody for 1 h at RT (BioLegend, Fell, Germany), followed by detection with avidin-horseradish-peroxidase (eBioscience, Frankfurt, Germany) for 1 h at RT. The sections were then counterstained with haematoxylin/eosin (HE) and analysed by transmitted light microscopy with an Axioskop 2 microscope (Leica Microsystems, Wetzlar, Germany).

For NIRF microscopy, sections were stained with DAPI (4 µg/ml) and anti-SiglecF-680. For fluorescence microscopy of lungs from anti-SiglecF-680 injected mice, cryosections were stained with anti-mouse-eosinophilic major basic protein (EMBP) antibody (clone S-16, Santa Cruz, Heidelberg, Germany) followed by anti-Alexa Fluor 488 secondary antibody, and/or anti-mouse CD68 antibody (clone FA-11, Abcam, Cambridge, UK), followed by anti-rat-Alexa Fluor 555 secondary antibody (Life Technologies GmbH, Darmstadt, Germany). Images were acquired with a Leica CTR6000 fluorescence microscope equipped with a Leica DFC350FX camera.

### Statistical Analysis

Statistical analysis was performed with Past [18] using a Welch t-test. P-values <0.05 were considered significant.

### Ethics Statement

This study was carried out in strict accordance with the guidelines for the care and use of laboratory animals of the local ethics office of the University Medical Center Göttingen. This study was approved by the Committee on the Ethics of Animal Experiments of the Niedersächsisches Landesamt für Verbraucherschutz und Lebensmittelsicherheit (LAVES) (Permit Number: 33.9-42502-04-10/0134). All painful procedures were performed under anesthesia, and all efforts were made to minimize suffering.

## Results

### Siglec-F is a Suitable Marker of Eosinophilia

**Figure 2:**
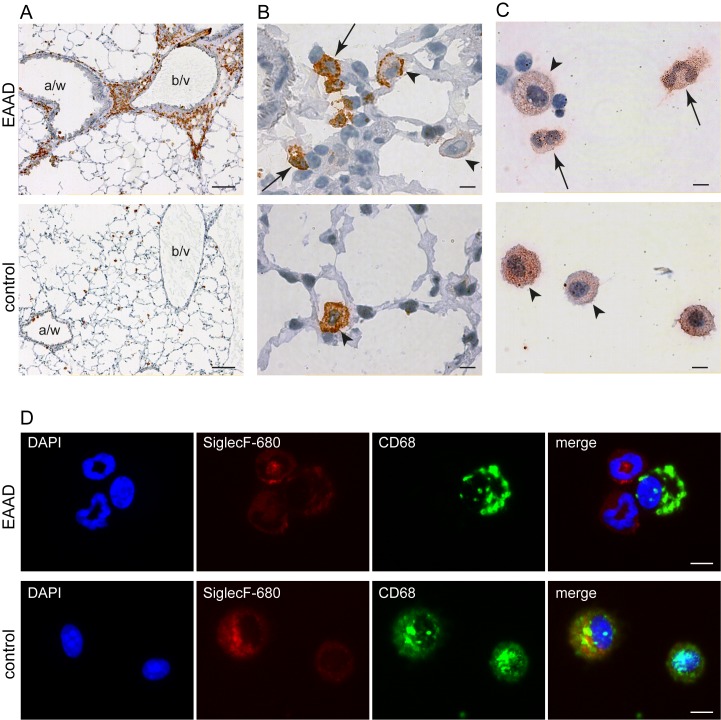
Expression pattern of Siglec-F. Immunohistochemistry and immunofluorescence Siglec-F staining of lung sections and BAL cytospins of mice with EAAD (A - D, upper panels) and controls (A – D, lower panels). (A)– (B) represent sections of cryofrozen lungs stained with anti-Siglec-F antibody. (C) Representative images of cytospins from BAL stained with anti-Siglec-F antibody and (D) of cytospins from BAL fluid co-stained with anti-SiglecF-680 and anti-CD68. In EAAD lungs, Siglec-F is highly expressed in eosinophils surrounding the blood vessels (b/v) and airways (a/w) (A, upper panel), while control lungs are almost free of Siglec-F staining (A, lower panel), indicating the lack of immune cell infiltration. Higher magnification of EAAD lung sections demonstrates Siglec-F staining on eosinophils (arrows, bilobed nucleus) and macrophages (arrow heads) (B, upper panel). In cytospins, eosinophils (bilobed nucleus) from EAAD animals (C and D upper panel, arrows) demonstrate strong positive Siglec-F staining, whereas macrophages from both, EAAD and control animals (C, arrow heads and D, positive CD68 staining) show a variety of Siglec-F expression levels. Scale bars in A: 2.5 mm; in B–D: 5 µm.

We first analysed the expression of Siglec-F on cells that accumulated in lungs of EAAD mice that did not receive the NIRF-labeled probe. Immunostaining of lung cryosections with an anti-Siglec-F antibody demonstrated a positive staining of cells that infiltrated the lung around blood vessels and airways of EAAD mice (Figure 2A, upper panel). However, only scattered cells within the lung tissue of control animals were Siglec-F positive (Figure 2A, lower panel). At higher magnification (100x, Figure 2B, upper panel), the positive cells in inflamed lungs are revealed to be mostly eosinophils as judged by their bilobed nuclei (arrows), and display a strong staining. A smaller number of weaker stained macrophages (large cells with unsegmented nuclei, arrow heads) were also detected in EAAD lung tissue. In control lung tissue the Siglec-F positive cells were all macrophages, as judged by their size and unsegmented nucleus (Figure 2B, lower panel, arrow head). As shown in Figure 2C, upper panel, immunostaining of cytospins from BAL fluid of EAAD mice with anti-Siglec-F antibody also revealed strong expression of Siglec-F on eosinophils, as confirmed by morphological appearance (cells with bilobed nuclei, arrows). Macrophages (larger cells, with unsegmented nuclei) revealed a variable degree of Siglec-F expression in both EAAD and control BAL (Figure 2C, arrow heads).

We then tested the two NIRF-labeled Siglec-F antibodies, anti-SiglecF-750 and anti-SiglecF-680, to confirm that these antibodies too, detect eosinophils and macrophages. For this purpose, we performed immunostaining of cytospins from BALs of EAAD and control mice. To better distinguish between cell types, we counterstained the nuclei with DAPI and labeled with anti-CD68, a common macrophage marker. We found that both NIRF-labeled antibodies were able to detect Siglec-F on eosinophils and macrophages. Figure 2D representatively shows the results for anti-SiglecF-680. In general, we found that macrophages with a high expression of CD68 also tended to exhibit a strong expression of Siglec-F.

### *In vivo* Detection of Siglec-F Expression

**Figure 3:**
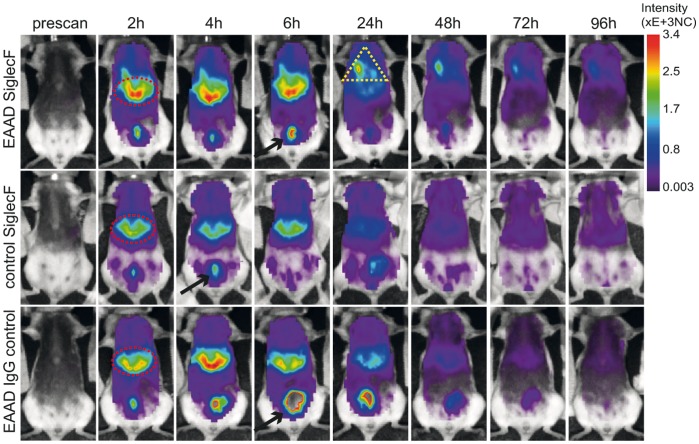
Time course of NIRF-labeled anti-Siglec-F distribution in the body. *In vivo* representative full body scans of EAAD (upper panel, n = 8) and control (middle panel, n = 6) mice injected with 12 µg of anti-SiglecF-750, as well as EAAD mice injected with 12 µg of Alexa 750-labeled anti-IgG2a isotype control antibody (lower panel, n = 5) at the given time points. Fluorescence intensity distribution is displayed in normalized counts (NC). Excess anti-SiglecF-750 antibody accumulates within the liver (red elipse) and is excreted via the bladder (black arrows) within the first few hours after antibody administration. 24 h –48 h after anti-SiglecF-750 injection, EAAD mice, in contrast to all control animals, accumulate the Siglec-F-antibody in their lungs (yellow triangle).

To assess the suitability of anti-Siglec-F-antibody for *in vivo* detection of EAAD we first conjugated it to Alexa Flour 750, a near infrared fluorescence dye with an excitation maximum at 750 nm. In this spectral range tissue autofluorescence is reduced due to minimal excitation of skin and hemoglobin, which are the main causes of autofluorescence. This approach improves signal-to-background ratio and hence the limits of detection. We then injected 12 µg of anti-SiglecF-750 i.v. into the tail veins of EAAD or control mice. As a negative control, EAAD mice were injected i.v. with an equal quantity of Alexa Fluor 750-labeled IgG2a isotype antibody at the same time point. Mice were scanned *in vivo* before (prescan) and after antibody injection for 4 days (24 h, 48 h, 72 h and 96 h) to determine the distribution of the antibody in the whole body. Figure 3 shows representative images of full body scans of an EAAD anti-SiglecF-750 injected mouse (upper panel), a control anti-SiglecF-750 injected mouse (middle panel) and an EAAD mouse which received the isotype control antibody (lower panel) at the given time points. Within the first 6 hours after antibody administration, excess antibody accumulated within the liver (red ellipses in Figure 3) and was excreted via the bladder in all mice (black arrows in Figure 3). At 24 h, most of the fluorescence signals over the liver and bladder area were cleared from all mice, which was similar in EAAD and healthy mice as well as in mice treated with the IgG2a control antibody. However, after 24 h, EAAD mice accumulated anti-SiglecF-750 in their lungs (yellow triangle in Figure 3, upper panel). This signal was detectable over the following 2 days and decreased to background levels at 96 h. In contrast, none of the control animals showed a specific signal within the lung at any time point (middle and lower panel).

To analyse the signals from the lungs in more detail, we performed *in vivo* lung scans by choosing a region of interest (ROI) over the lungs of the animals. These were performed with an increased raster resolution (1 mm) as well as higher integration time (1 s). To verify that the measured fluorescence intensities originated from the injected conjugates, we performed a lifetime analysis and compared the results with the lifetime of the pure conjugate. The lifetime of fluorescence signals measured *in vivo* over the lung region was the same as that of the pure conjugate measured *ex-vivo* (0.85 ns for anti-SiglecF-750 and 1.52 ns for anti-SiglecF-680) and was substantially higher than the lifetime of the autofluorescence background measured in the prescan over the same region (0.33 ns at 750 nm and 0.63 ns at 680 nm) (data not shown).

**Figure 4:**
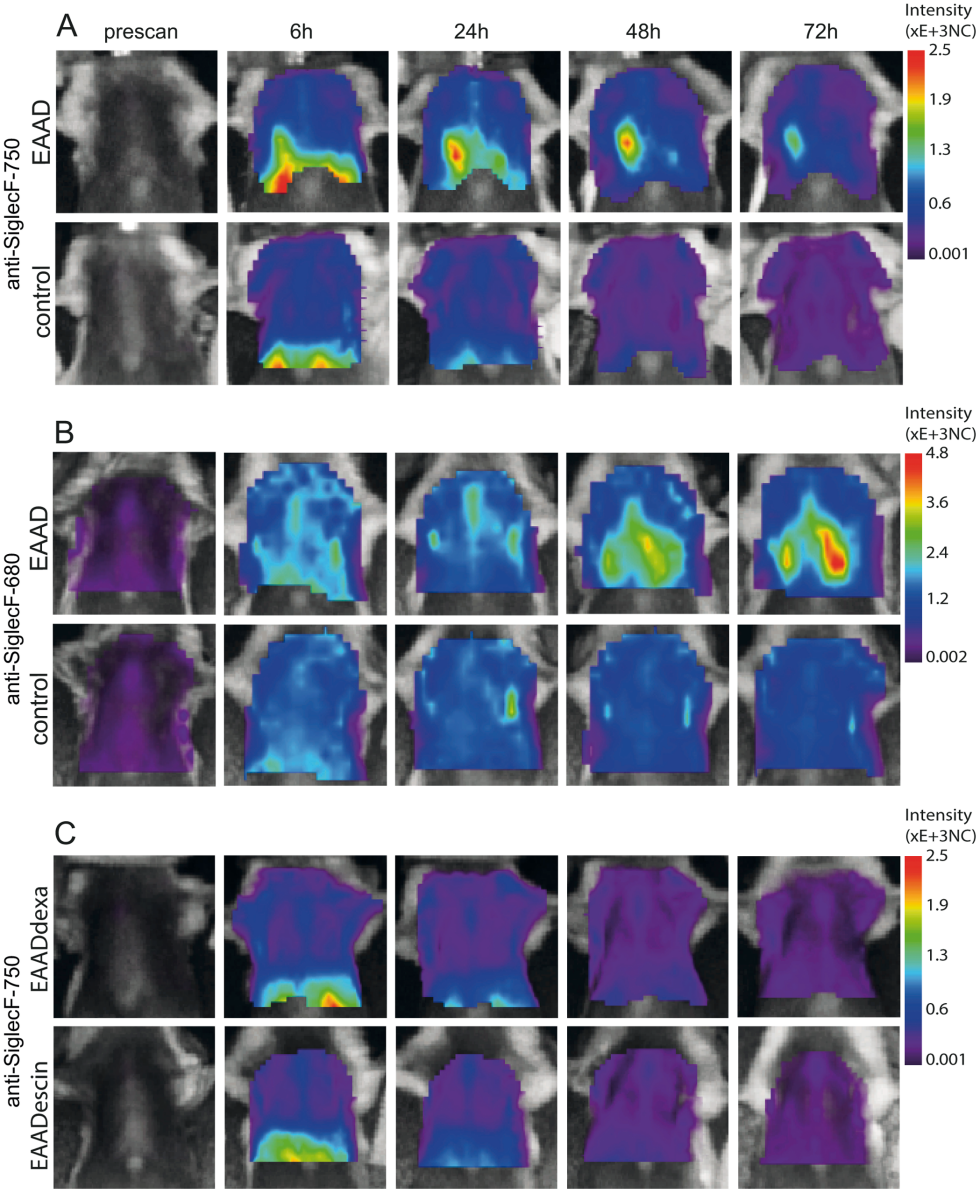
Time course of NIRFlabeled anti-Siglec-F distribution in the lung. *In vivo* lung scans of EAAD, control as well as dexamethasone and beta-escin treated animals before (prescan) and at 6 h, 24 h, 48 h and 72 h after antibody administration. Fluorescence intensity distribution is displayed in normalized counts (NC). In contrast to control mice (A, lower panel, n = 6), OVA-immunized mice have a marked accumulation of anti-SiglecF-750 within the lungs from 24 h, which decreases at 72 h (A, upper panel, n = 8). Anti-SiglecF-680 also reveals significant differences between EAAD (B, upper panel, n = 5) and control (B, lower panel, n = 4) fluorescence intensities derived from the lung. EAAD mice treated with either dexamethasone (C, upper panel, n = 5) or beta-escin (C, lower panel, n = 5) have low intensities over the lung, similar to healthy control mice (A and B, lower panels) at all scan times.

**Figure 5:**
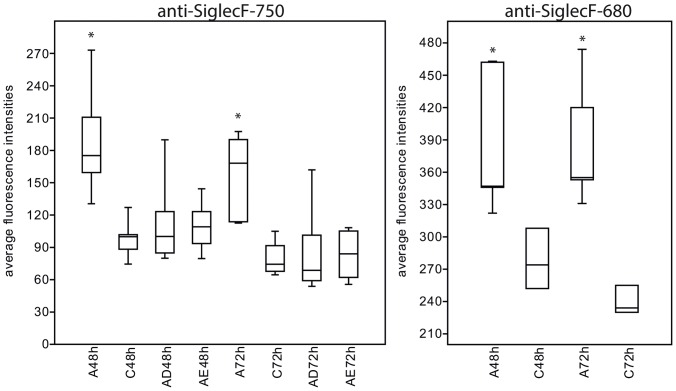
Quantification of *in vivo* imaging results. Box plot of average fluorescence intensities over the lung area for all groups at 48-labeled anti-Siglec-F antibody injection. Lung intensities of EAAD mice are significantly higher (represented by asterisk *) compared with control mice and treated mice at 48 h and 72 h after antibody application; A = EAAD, C = control, AD = EAAD, dexamethasone treated, AE = EAAD, beta-escin treated.

Figure 4A shows representative images of lung scans of OVA-induced EAAD mice and healthy control mice before and 6 h, 24 h, 48 h and 72 h after anti-SiglecF-750 application. During the first 6 h, low fluorescence intensities were detected over the lungs in control and EAAD animals, which was probably, in part, due to the scattering of signals originating from the liver. This low signal decreased over the next 3 days in control mice (Figure 4A, lower panel). In contrast, OVA-immunized mice had a high fluorescence signal over the lung indicating an accumulation of NIRF-labeled antibody within the lungs over the following 3 days (Figure 4A, upper panel). Mice with EAAD exhibited a 1.4 (24 h scan, p = 0,04) –2.0 (48 h scan, p = 0,00035 and 72 h scan, p = 0,00012) times higher anti-SiglecF-750 signal over the lungs compared to control animals (Figure 5, left panel). The presence of EAAD was confirmed by HE staining of lung cryosections at the end of the experiment (96 h after antibody administration), which demonstrated immune cell infiltration around the bronchi and vessels within the lung (data not shown).

To verify the results with a dye with a higher quantum yield, we used an anti-Siglec-F antibody coupled to Alexa Fluor 680 (anti-SiglecF-680) in the same EAAD model and measured the signal intensities over the lung at the altered wavelength of 680 nm in EAAD and control animals. All other conditions and time points were unchanged. Figure 4B shows representative images of one animal illustrating that anti-SiglecF-680 also leads to high signal intensities in the lungs of EAAD mice (upper panel), while control animals displayed much lower signals (lower panel). Anti-SiglecF-680 signal intensities were 1.4 (48 h scan, p = 0,01) –1.6 (72 h scan, p = 0,0021) times higher in lungs of EAAD mice when compared to controls (Figure 5, right panel). Notably, the intensities with anti-SiglecF-680 were generally higher than those with anti-SiglecF-750, which was mainly due to differences in the quantum yields of the dyes (0.12 for Alexa Fluor 750 versus 0.36 for Alexa Fluor 680; www.lifetechnolgies.com), as the difference between the dye to protein ratios were already accounted for.

Peak average intensities of lung signals in animals with EAAD were observed across the range of detection time points, with 7 animals showing maximum intensity 24 h after antibody application, 1 exhibiting a maximum signal 48 h, and 5 with maximum signal 72 h after anti-Siglec-F-750 or anti-SiglecF-680 injections. This is indicative of a difference in the progress of acute onset or resolution of EAAD with a varying accumulation of eosinophils and other infiltrating immune cells in the lungs. However, by 96 h, all animals with acute EAAD had reduced specific fluorescence signals over the lungs.

Out of 13 OVA-immunized mice, only two displayed a homogenous fluorescence signal distribution over the lung, whereas all others showed an asymmetrical distribution of signal intensities (Figure 4A and 4B). Eight mice showed a higher signal on the right side of the lung, while 3 mice demonstrated more signal on the left side, reflecting differential cellular infiltration in different lung lobes, based on histological examination of lung tissue from EAAD mice (data not shown). In summary, fluorescence intensities over the lungs of EAAD mice *in vivo* were significantly higher compared to those of control mice at 48 h and 72 h after antibody application, as demonstrated in the box plot (Figure 5).

### *Ex vivo* Confirmation of Siglec-F Expression

**Figure 6:**
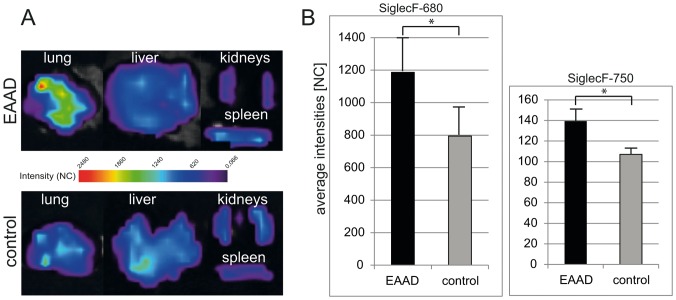
*Ex vivo* imaging results. (A) Representative images of fluorescence intensities of explanted lungs, livers, kidneys and spleens of EAAD (upper panel) and control mice (lower panel). (B) Bar graph of average fluorescence intensities of explanted lungs from mice injected with anti-SiglecF-680 (left panel) or anti-SiglecF-750 (right panel). *Ex vivo* lung scans demonstrate a significant difference between signal intensities of EAAD lungs and healthy lungs (A and B), while liver, spleen and kidneys show low intensities in both EAAD and control mice (A). NC = normalized counts.

To verify that the *in vivo* measured signals originated from the lung, we performed scans of the excised lungs from animals sacrificed after the last *in vivo* scan, seen in Figure 6A. *Ex vivo* scans confirmed that lungs from EAAD mice displayed a significantly higher fluorescence signal intensity than control animals up to 96 h after i.v. injection of anti-SiglecF-750 (1.3 times higher in EAAD, p = 0,007) (Figure 6B, right panel) or anti-SiglecF-680 (1.5 times higher in EAAD, p = 0,03) (Figure 6B, left panel). Note, that here too, the average intensities with anti-SiglecF-680 are higher than with anti-SiglecF-750, due to the higher quantum yield of Alexa Fluor 680.

Other organs such as liver, kidneys and spleen showed substantially lower Siglec-F signals in both EAAD and control animals (Figure 6A). Signals in liver and kidneys of control mice were generally somewhat higher than in the same organs of EAAD mice, which may be explained by a higher amount of unbound NIRF-labeled anti-Siglec-F antibody that is cleared by the liver and kidneys.

**Figure 7:**
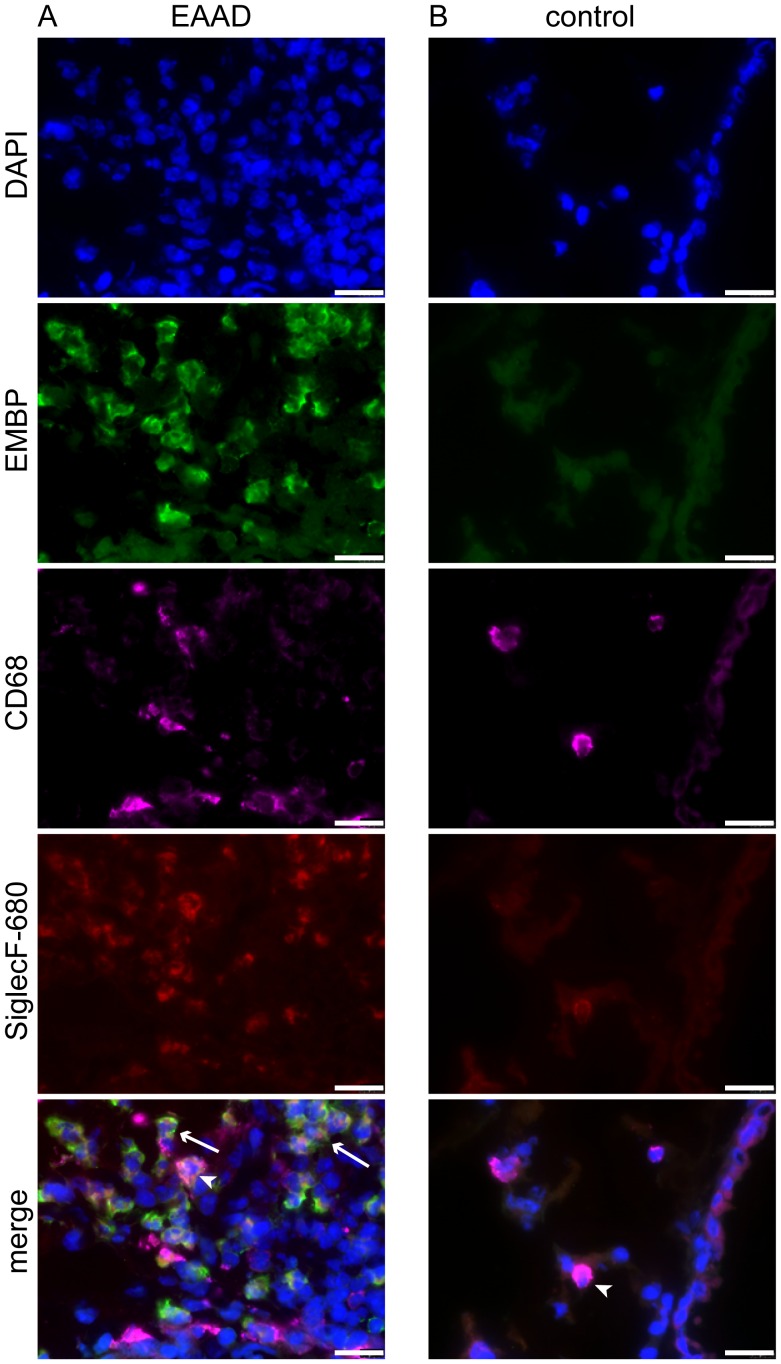
Anti-SiglecF-680 binds to eosinophils and macrophages. Fluorescence microscopy of cryosections from lungs of EAAD mice injected with anti-SiglecF-680 (A), confirms the binding of anti-SiglecF-680 (in green) to eosinophils (EMBP-positive, arrows in merge) and more weakly to macrophages, which were counterstained with anti-CD68 (magenta, arrow heads in merge). Lungs from healthy controls injected with anti-SiglecF-680 (B) have a low number of Siglec-F positive cells, which are all CD68-positive and therefore most probably represent macrophages. Nuclei are stained blue with DAPI. Scale bars = 20 µm.

To analyze the binding of the injected anti-SiglecF-680 to lung cells in more detail, frozen tissue sections were visualized with a fluorescence microscope. To better distinguish between eosinophils and macrophages, lung sections were stained with an antibody directed against EMBP (eosinophilic major basic protein), an eosinophil marker and anti-CD68 antibody, a macrophage marker. Nuclei were visualized with DAPI. As seen in Figure 7A, anti-SiglecF-680 was mainly bound to eosinophils around bronchi and vessels in EAAD lungs (EMBP-positive, arrows in merged image), but was also present on some macrophages (CD68-positive, arrow head in merged image). Co-localization of anti-SiglecF-680 (red) and anti-EMBP (green) resulted in a yellow staining of eosinophils in the merged image. Healthy control lungs displayed few anti-SiglecF-680 positive cells (Figure 7B), which were all CD68 positive (arrow in merged image) and therefore, macrophages and demonstrated vesicle-like Siglec-F staining in the cytoplasm. These results confirm that the higher *in vivo* signals we found in EAAD mice derive mostly from anti-SiglecF-NIRF antibody bound to eosinophils.

### *In vivo* Monitoring of EAAD in Response to Therapy

To investigate the feasibility of anti-SiglecF-750 to assess the therapeutic response of mice with EAAD *in vivo*, we performed lung scans after treatment with dexamethasone or the natural compound beta-escin, as indicated in the Methods section in Figure 1. Both dexamethasone- and beta-escin-treated mice showed comparable signal intensities over the lungs that were similar to healthy control mice (Figure 4C) and significantly different to untreated EAAD animals at 48 h and 72 h after antibody application (Figure 5, left panel). Average lung intensities of dexamethasone-treated mice were 1.6 (p = 0,01) and 1.8 (p = 0,0059) times lower than untreated EAAD lungs at 48 h and 72 h, respectively. Average lung intensities of beta-escin-treated mice were 1.8 (p = 0,0034) and 2.0 (p = 0,00079) times lower than in untreated EAAD mice at 48 h and 72 h after antibody injection. In dexamethasone-treated mice, the response to treatment, as evaluated by HE staining of lung cryosections from mice sacrificed 96 h after antibody administration, correlated with *in vivo* signal intensities. Mice that showed a low amount of cell infiltration and decreased bronchial wall thickness due to successful therapy, also had low fluorescence signals over the lung (Figure S1, C–D). Despite therapy with dexamethasone, 2 mice still had inflammation around bronchi and vessels and showed fluorescence signals over the lung above control levels (supplementary Figure 1, A–B). These results demonstrate that the reduction of lung cell infiltration upon treatment could be successfully monitored non-invasively by NIRF imaging with the anti-SiglecF-750 probe (Figure 4C, lower panel). In summary, both dexamethasone and beta-escin treatment reduced anti-Siglec-F-750 lung signal intensity in EAAD mice to that of healthy controls.

## Discussion

Here, we show that the antibody targeting Siglec-F is a novel NIRF probe that can be applied for non-invasive optical imaging of EAAD in mice, during the course of the disease as well as in response to therapy. We found that upon administration of anti-SiglecF-750 and anti-SiglecF-680 probes there were significantly higher fluorescence signals in the lungs of mice with EAAD compared to healthy controls, demonstrating that NIRF imaging was successfully used to distinguish EAAD and healthy mice *in vivo*.

FRI-NIRF-based studies of the lung have, to date, found little use in preclinical *in vivo* studies. However, a recent study demonstrated the application of a novel NIRF-labeled probe containing dendritic polyglycerol sulfates to monitor inflammation in OVA-induced EAAD in mice, by targeting selectins [11]. Furthermore, the enzyme-based and commercially available activatable probes, MMPSense and ProSense (PerkinElmer) were shown to be useful for detection of lung inflammation [9], [10], [19]. These probes are optically silent in their inactivated state, but become fluorescent following activation by either matrix metalloproteinases (MMPs) or cathepsins. A concern is that these probes are not specific for lung inflammation, and might target inflammation elsewhere. Additionally, smart-probes activated by cathepsins and MMPs do not directly bind any target, leading to a high background from unbound probe. These factors do not apply for anti-Siglec-F antibody, as it binds directly to eosinophils. Since eosinophilia is one of the hallmarks of allergic asthma, Siglec-F represents a useful marker for disease imaging of EAAD.

In support of previously published studies [13], [20], we found a pronounced expression of Siglec-F on lung eosinophils of EAAD mice. Furthermore, the binding of the i.v. applied antibody to eosinophils was verified by NIRF microscopy on EAAD lung sections. We administered anti-Siglec-F at the peak of eosinophilia, which in this model is 3–4 days after the last i.n. challenge [21], [22], to achieve a high expression level of Siglec-F within the lung that could be detected by the NIRF-labeled antibody. Notably, several studies have shown that Siglec-F may play a role in the resolution of the acute allergic reaction by inducing eosinophil apoptosis [13], [20], [23]. For instance, in mice lacking Siglec-F, there is delayed resolution of lung eosinophilia and reduced peribronchial cell apoptosis in a model of EAAD [13]. In addition, the administration of an anti-Siglec-F antibody significantly reduced allergen-induced eosinophilic airway inflammation owing to the reduced production of eosinophils and an increase in apoptotic eosinophils in lung, blood, and bone marrow [13], [20], [23]. Based on these studies, anti-Siglec-F antibody has been considered as a therapeutic drug for eosinophilic disorders including acute and chronic asthma [20], [24], [25]. To avoid the potential anti-Siglec-F antibody-induced eosinophil apoptosis effect on the course of disease, we administered the antibody 4 days after OVA challenge. However, we cannot rule out that the decline of the signal detected in EAAD mice within 24–48 h of receiving the antibody was caused by anti-Siglec-F-induced apoptosis of eosinophils. We would argue that the decline of the fluorescence signal is mostly likely due to degradation or resorption of the antibody or a result of the normal physiological resolution of allergic inflammation. Nevertheless, it is tempting to speculate that anti-Siglec-F antibody could be used as a combined diagnostic and therapeutic tool for allergic eosinophilic inflammation for preclinical animal models.

*Ex vivo* results indicate that the anti-Siglec-F antibody not only targets eosinophils, but also binds to macrophages around the vessels and bronchioles of the EAAD lung tissue, which supports previous findings [14]. Because macrophages increase in number in the lungs of mice with EAAD, it is likely that the increased Siglec-F signal in EAAD mice as well as the low but measurable signal in control mice originated, in part, from the antibody binding to macrophages. Furthermore, macrophages are involved in the phagocytosis of apoptotic eosinophils during the resolution of eosinophilia [26], [27], and probably the underlying mechanism for the observed Siglec-F expression in vesicle-like structures within macrophages from EAAD mice.

Even though anti-Siglec-F antibody does not bind exclusively to eosinophils and plays a role in eosinophil apoptosis, our results show that it can be used as a probe to distinguish allergic lung inflammation from healthy lung. There are additional eosinophil markers, such as CCR3, EMBP, CD23, CD48, and CD147, which are considered useful as potential *in vivo* targeting probes, but they also bind to other immune cells, such as mast cells or Th cells [28]–[31], and may affect the course of the disease to a larger degree than anti-Siglec-F.

Our choice to utilize NIRF imaging for these studies was based on several advantages over imaging methods such as MRI and PET. One advantage is that there is a low signal-to-noise ratio and high tissue penetration [32]. NIRF imaging using commercially available dyes, Alexa Fluor 680 and Alexa Fluor 750 [33] coupled to Siglec-F antibody, were highly suitable for distinguishing between EAAD and controls. The fluorescence signals we detected *in vivo* over the lung of EAAD mice were specific, as an isotype control antibody did not demonstrate fluorescence intensities above controls. Furthermore, the lifetime of these signals was the same as that of the pure probe and substantially higher than the lifetime of the autofluorescence background measured in prescans. We observed that Alexa Fluor 680, in general, exhibited higher fluorescence intensities, which was probably due to its higher quantum yield. However, this resulted in higher background levels and may have contributed to the lower signal ratios observed between EAAD and control mice injected with anti-SiglecF-680. The majority of EAAD mice showed a difference in signal intensity between the right and left lung, which reflects the distribution of inflammation in the lungs based on histological examination of lung tissue from EAAD mice. Our findings are supported by previous studies demonstrating heterogeneous distribution of allergic inflammation in the lung using CT and MRI [34] and in a study of non-invasive optical tomography using NIRF-labeled smart probes [10].

Not only could normal, healthy lungs be distinguished from mice with allergic lung inflammation, but it was possible to monitor therapeutic efficacy with our anti-Siglec-F imaging approach. EAAD mice treated with glucocorticosteroids or beta-escin exhibited reduced eosinophilic inflammation, as visualized by the NIRF-labeled anti-Siglec-F antibody. Treatment with dexamethasone and beta-escin reduces lung eosinophilia [35], [36] and in our experiments led to low intensity signals over the lungs of EAAD mice that were similar to healthy controls. These data confirm the suitability of anti-Siglec-F-NIRF as a method not only for detecting allergic lung inflammation but also for monitoring the efficacy of novel therapies for the treatment of EAAD in preclinical *in vivo* studies.

A feature that distinguishes EAAD from human asthma is the level of airway eosinophilia. The model used in these studies leads to approximately 30% eosinophils in the airways [22], which is higher than airway eosinophilia in humans. Nevertheless, this model is optimal for proof of concept for our imaging approach, because untreated EAAD mice have significantly higher lung eosinophilia compared with treated and healthy mice [37], which allows the differences between healthy, treated, and untreated groups to be easily distinguished. We would argue that our approach works well in small animals and could be used effectively in preclinical models. However, there are some limitations using this approach clinically. Recent studies demonstrate that Siglec-F functions differently in animals and humans. For example, Mao et al. showed, that Siglec-F-mediated apoptosis differed in magnitude and underlying mechanism in mice compared to Siglec-8-mediated human eosinophil apoptosis [38], which may be explained by the fact that Siglec-8 is a functional paraloq of Siglec-F, rather than a true ortholog. Moreover, other targets that were successfully used therapeutically in EAAD were ineffective in humans [30], [39]. This indicates that the differences between EAAD and human asthma in regard to eosinophilia, T-cell response and AHR need to be considered [15] and entail optimization of both animal models and probes. Therefore, the use of anti-Siglec-F/Siglec-8 antibody as a theranostic tool for human asthma will require further study.

### Conclusion

In conclusion, we demonstrate the suitability of NIRF-labeled anti-Siglec-F-antibody probe in combination with FRI imaging as a novel tool for non-invasive monitoring of allergic lung inflammation in mice, for monitoring treatment efficacy and progression of other eosinophil-related diseases.

## Supporting Information

Figure S1 ***In vivo*** **imaging results of treated mice correlate with peribronchial inflammation.** The upper panel represents 48 h scans of 5 different EAAD mice treated with dexamethasone. Samples (A) and (B) demonstrate a low but measurable anti-SiglecF-750 signal in the lung (arrow heads) in comparison to (C)-(E). The corresponding HE staining of lung cryosections at the end of the experiment (lower panel) shows the two samples with fluorescence signal have remaining cell infiltration (arrows) despite therapy. All other samples reveal a complete resolution of inflammation, as judged by the lack of infiltrating immune cells.
(TIF)
