# Supplementary figures and images for: Non-Invasive Optical Imaging of Eosinophilia during the Course of an Experimental Allergic Airways Disease Model and in Response to Therapy
Source: PLoS One. 2014 Feb 25;9(2):e90017. doi: 10.1371/journal.pone.0090017 (PMC3934967; doi:10.1371/journal.pone.0090017)

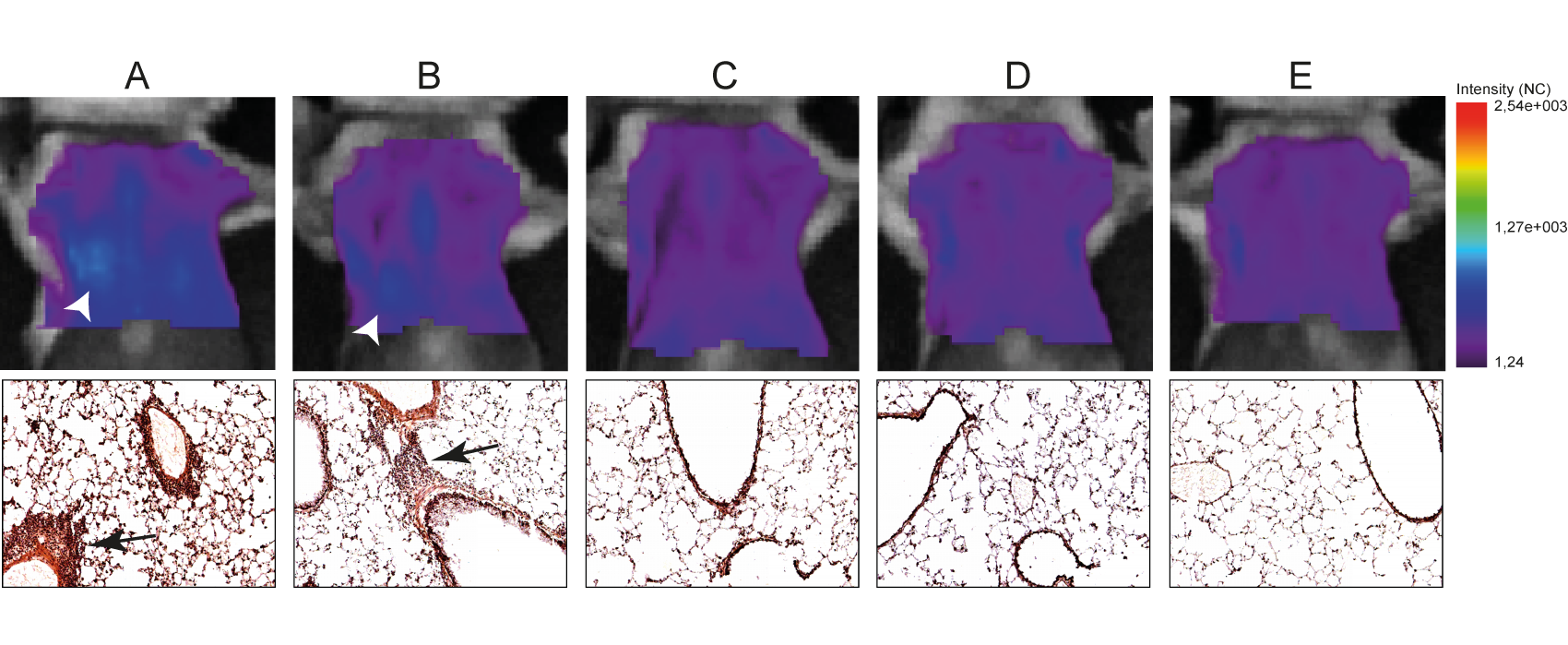

Supplement: Figure S1 — In vivo imaging results of treated mice correlate with peribronchial inflammation. The upper panel represents 48 h scans of 5 different EAAD mice treated with dexamethasone. Samples (A) and (B) demonstrate a low but measurable anti-SiglecF-750 signal in the lung (arrow heads) in comparison to (C)-(E). The corresponding HE staining of lung cryosections at the end of the experiment (lower panel) shows the two samples with fluorescence signal have remaining cell infiltration (arrows) despite therapy. All other samples reveal a complete resolution of inflammation, as judged by the lack of infiltrating immune cells. (TIF) [file pone.0090017.s001.tif]
